# High-Cadence Cycling Promotes Sustained Improvement in Bradykinesia, Rigidity, and Mobility in Individuals with Mild-Moderate Parkinson's Disease

**DOI:** 10.1155/2019/4076862

**Published:** 2019-03-03

**Authors:** Angela L. Ridgel, Dana L. Ault

**Affiliations:** Department of Exercise Physiology, Kent State University, 350 Midway Dr., Kent, OH, USA

## Abstract

**Introduction:**

Exercise has been shown to be an important adjunct therapy to medication in Parkinson's disease (PD). However, the optimal type, frequency, and intensity of exercise or physiotherapy are still being debated. An important part of understanding the optimal frequency is to examine how acute bouts of exercise affect motor function and mobility in this population. The purpose of this study is to assess if six bouts of high-cadence cycling improves motor function and mobility in individuals with PD.

**Methods:**

Sixteen subjects with mild-moderate idiopathic PD were randomized into either a high-cadence cycling or a control (stretching) group. High-cadence cycling was completed on a custom motorized recumbent bicycle at a high cadence between 75 and 85 rpm. Cycling and stretching sessions were separated by 1 day of rest and took place over a 15-day period. Motor function and mobility were assessed after every cycling/stretching bout using the UPDRS Motor III scale, Kinesia ONE, and Timed up and Go (TUG).

**Results:**

Six bouts of high-cadence cycling improved UPDRS scores (2.5 pts, *P*=0.002), hand movement amplitude (*P*=0.013), rapid alternating hand movement speed (*P*=0.003), gait (*P*=0.012), and TUG time (1.17 s, *P*=0.002) from baseline testing to end of treatment. The control group showed no improvements.

**Conclusions:**

These findings suggest that they are both acute and sustained improvements in motor function and mobility after high-cadence cycling. Future research should examine how exercise type, frequency, and intensity can be optimized for each individual.

## 1. Introduction

More than 600,000 people in the US have been diagnosed with Parkinson's disease (PD), and the prevalence of this disease is predicted to increase to over 1 million by 2030 [1, 2]. Several recent studies have shown that exercise can be beneficial in improving walking, balance, and muscle strength and the performance of activities of daily living in individuals with PD [3–9]. It has been suggested that activity-dependent neuroplasticity can be driven by high speed, complex goal-directed exercise (high velocity, complexity, and repetition) [10–17]. Fisher and colleagues showed that high-velocity treadmill training altered cortical inhibitory mechanisms, as measured with transcranial magnetic stimulation, and promoted improvements in gait in individuals with PD [16]. High-cadence tandem cycling reduced PD symptoms in both the upper and lower extremity and increased brain activation as measured by fMRI [10, 13, 14]. Furthermore, three sessions of high-cadence dynamic cycling on a motorized stationary cycling improved UPDRS Motor III scores more than individuals cycling at a low cadence. These finding suggest that the high velocity and complexity of these different types of training was the driver of these changes.

However, there are still several unanswered questions such as (1) what is the optimal frequency of training, (2) how long do the benefits last after a session or a series of sessions, and (3) which motor symptoms of PD are most affected by high-velocity training. In addition, few studies have examined how training alters the quality of movement. In most exercise intervention studies, UPDRS Motor III scores are used to assess motor function; however, these scores do not quantitatively examine the quality of movement. Therefore, the primary aim of this study was to examine acute changes in motor function and mobility in individuals with PD after bouts of high-cadence cycling and to assess if changes were maintained 48 hours after the last cycling session. We hypothesize that six bouts of high-cadence dynamic cycling would promote sustained improvement in PD motor symptoms, motor function, and mobility in individuals with PD. A secondary aim was to examine how this training alters the quality of movement. This work builds on previous studies [13, 18, 19] by providing data on both immediate and sustained (after 6 sessions) benefits of high-cadence cycling.

## 2. Methods

### 2.1. Participants

Individuals with idiopathic Parkinson's disease were recruited from support groups, local Parkinson's symposium, and from a list of individuals who previously participated in studies in our lab. Inclusion criteria included a diagnosis of idiopathic PD, Hoehn and Yahr scale 1–3, 50–79 years of age, and no contraindications to exercise including untreated cardiovascular disease or stroke (Table 1). The exclusion criteria also included individuals who were identified as “high risk” for a cardiovascular event (AHA/ACSM Guidelines) and individuals with unpredictable motor fluctuations. Participants were instructed to maintain their current physical activity level and medication schedule and were assessed in the “on” medication state. All participants obtained a clearance from their physician prior to testing and signed written informed consent. The study was approved by the Institutional Review Board (IRB) at Kent State University (IRB #15-544, November 2, 2016 to November 1, 2017) was conducted in accordance with the Belmont Report.

### 2.2. Protocol

**Figure 1:**
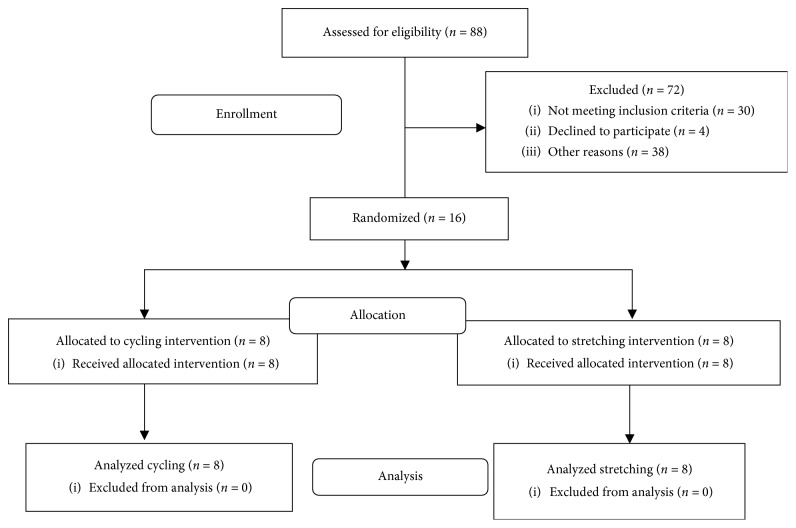
Consort diagram. This diagram shows the recruitment, randomization, and data collection process of this study.

All eligible participants were asked to visit the exercise physiology laboratory for seven sessions over a 15-day period (Figure 1). Each subject started on a Thursday and sessions continued with a Tuesday/Thursday/Saturday schedule. On the first visit, participants were randomized into the cycling or stretching group and were given a wrist-based activity tracker [20] (Moveband 2, DHS, LLC, Cleveland, OH) to track their physical activity levels (steps per day) over the course of the study. During the first session, participants completed assessment tests (UPDRS Motor III, Kinesia ONE, Timed Up and Go), performed the designated intervention (dynamic cycling or stretching), and then completed the same assessment tests within 5 minutes of completing the intervention. During sessions 2–6, participants completed their designated intervention before completing the motor function assessment tests. On session 7 (48 hrs after the last cycling/stretching session), participants only completed the assessment tests. Additional details on the protocol can be found in Ault [21].

### 2.3. Intervention

Participants in the dynamic cycling completed six 40-minute sessions of dynamic cycling. High-cadence dynamic cycling is described in a previous study [13]. In short, the intervention consisted of a 5-minute warm-up on the customized motorized, stationary cycle at 50 revolutions per minute (rpm), 30-minute of high-cadence cycling, and 5-minute cool-down at 50 rpm. During the 30 minutes of high-cadence cycling, the motor speed was set for 80 rpm. Participants were informed that the bicycle motor would provide assistance but that they would have to push on the pedals. If individuals were able to keep up with the motor or pedal faster than 80 rpm motor, then torque values would be positive. If participants were not able to achieve 80 rpm (pedaling slower than 80 rpm), then negative torque indicated that the bicycle motor was performing more work than the participant. Participants were encouraged to display positive torque numbers on the control box. During high-cadence cycling, the heart rate, rating of perceived exertion (RPE), cadence, and torque were recorded. The heart rate was measured with a chest strap (Polar T31); RPE was assessed with the 6–20 Borg scale [22]; and a computer connected to the control box of the dynamic cycle recorded cadence and torque. Assessments were completed approximately five minutes after each cycling session.

Participants in the control group also completed six 40-minute sessions, which included a 5-minute warm-up on a Schwinn Airdyne bicycle and 35 minutes of seated upper and lower body stretches. All stretches were held for 20 seconds, and then a second round of stretches was completed. Stretches included head tilt forward, head tilt sideways (right/left), head rotation (right/left), arm-in-front shoulder stretch, upper-arm-up shoulder stretch, arms behind back reach, seated trunk rotation, seated side stretch, seated wall calf stretch, seated hamstring stretch, seated quad stretch, seated piriformis stretch (leg crossed over knee), seated knees to chest, outside ankle rotation, and inside ankle rotation. Assessment tests were completed approximately five minutes after each stretching session.

### 2.4. Assessment Tests

The Unified Parkinson's Disease Rating Scale (UPDRS) part III was used to assess motor symptoms and was administered by a research assistant who was trained using the Movement Disorders Society training video [23]. Although UPDRS III is a standard clinical assessment, we also evaluated tremor, bradykinesia, dyskinesia, mobility, and movement amplitude, rhythm, and speed using a device called Kinesia ONE (Great Lakes Neurotech, Cleveland, OH) to remove potential assessor bias. Kinesia ONE includes a wireless finger sensor and an iPad to display pictures of each motor function test, which prompted participants through each motor function test. Motor function test included measures of bradykinesia (finger and toe taps, opening and closing hand, palm rotation). To evaluate gait, the wireless sensor was placed on the shoe [24, 25] while the individual walked in a straight line and completed a half turn. Angular velocity and coefficient of variation of time during leg swing while walking and the rotation time and angular velocity to complete a half turn was analyzed. The wireless sensor transmitted information back to the iPad where an algorithm analyzed the information and provided unbiased scores on a 0 to 4 scale. This device has been shown to be highly correlated to UPDRS clinical scores [24].

Mobility of the participants was evaluated through the Timed Up and Go (TUG) test. Participants began in a seated position in a chair, and when the researcher said “go,” the participant got up from the chair and walked at a preferred speed for 3 meters, turned around a cone, walked back to the chair, and sat down. The researcher recorded the amount of time it took the participant to walk 3 meters and sit back down in the chair. Time started when the researcher said “go,” and time stopped when the participant sat back down in the chair. Participants completed two trials, and the average time of those trials was used for analysis. Time taken to complete the test is strongly correlated to the level of functional mobility [26].

### 2.5. Statistical Analysis

Using IBM SPSS Statistics 20 (SPSS, Inc., Chicago, IL, 2005), an independent *t*-test was done to compare demographic variables between the two groups. A 2 × 8 (group-by-session) repeated measures analysis of variance (ANOVA) was performed to determine differences between groups and sessions for the UPDRS Motor III. If interactions were present, then a paired sample *t*-test, with Bonferroni correction for multiple comparisons, was used to determine where differences were present. Statistical significance was set at *P* < 0.05. Pre- and postvalues of Kinesia ONE variables were analyzed in the dynamic cycling group using paired samples *t*-test. All data are presented as mean ± standard deviation (SD).

## 3. Results

Sixteen subjects (9 males and 7 females) with mild-moderate idiopathic PD were randomized into either a cycling or stretching group (Figure 1). Age, height, baseline UPDRS Motor III scores, and duration of PD were not significantly different between the two groups, but weight and Hoehn and Yahr scores were significantly different between groups (Table 1). The control group had a higher H&Y score than the cycling group. The difference in weight is likely due to the gender distribution (stretching group had five males). All participants in the dynamic cycling group were able to complete the sessions and cycled at an average of 64% (96 ± 12 bpm) of their age-predicted maximum heart rate (220 − age). Two individuals were on beta-blockers so their heart rate was not analyzed. Individuals cycled at an average cadence of 78.9 ± 2.3 rpm (range: 75.5–82.4) and average torque was 4.34 ± 11.32 (range: −11.1–19.2). None of these subjects had episodes of freezing during the study.

### 3.1. UPDRS Motor III

**Figure 2:**
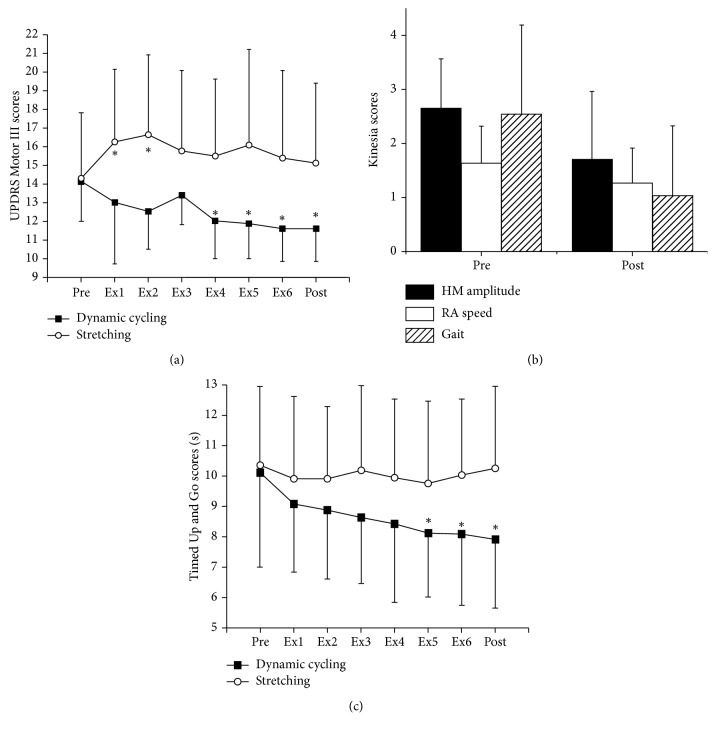
(a) UPDRS Motor III scores of high-cadence cycling and control groups from baseline testing (pre) to 48 hours after the last cycling session (post). The high-cadence cycling group showed a sustained and significant improvement in their UPDRS score from pretesting to posttesting. (b) Kinesia score for hand movement amplitude, rapid alternating movement speed, and gait showed significant improvements after high-cadence dynamic cycling. (c) Timed Up and Go (TUG) scores of high-cadence cycling and control groups from baseline testing (pre) to 48 hours after the last cycling session (post). ^*∗*^*P* < 0.05 compared to prescores.

There was a significant (*P* < 0.001) group-by-time interaction in the overall UPDRS Part III Motor scores (*F* = 5.82, df = 7, *P* < 0.001, Figure 2(a)). Bonferroni correction was set to *P* ≤ 0.006(0.05/8) to account for paired samples *t*-test comparison. Specifically, there were significant improvements in the high-cadence cycling group between pretesting and session 4 (*P*=0.001), session 5 (*P*=0.001), session 6 (*P*=0.002), and posttesting (48 hours after the last cycling session, *P*=0.002). UPDRS scores in the control group showed a significant worsening between pretesting and session 1 (*P*=0.004) and session 2 (*P*=0.004). A repeated measures ANCOVA analysis with Hoehn and Yahr scores as a covariate was also completed to account for the baseline differences in the two groups. The significant group by time interaction was maintained with the addition of this covariate (*F* = 4.57, df = 7, *P* < 0.001). An analysis of the UPDRS scores from pre to post in both groups showed that the high-cadence cycling group significantly improved by 2.5 points (17%, *F* = 20.05, df = 1, *P*=0.001) after 6 cycling sessions while the stretching group showed no significant change. We also analyzed the UPDRS scores based on symptoms type in order to determine which showed the greatest change after six bouts of dynamic cycling. There was a significant improvement between pre and post in rigidity (*t* = 2.393, df = 7, *P*=0.048), finger taps (*t* = 2.376, df = 7, *P*=0.049), hand movements (*t* = 3.416, df = 7, *P*=0.011), and pronation/supination (*t* = 2.393, df = 7, *P*=0.048). No other parts of the UPDRS Motor III showed improvement.

### 3.2. Bradykinesia and Gait Assessment

Kinesia ONE was used to examine of the quality of movement (speed and amplitude) during the finger tap, hand movement, and pronation/supination tests and during a gait assessment in the dynamic cycling group only. In the dynamic cycling group, there was a significant improvement in hand movement amplitude by 36% (*t* = 1.62, df = 7, *P*=0.013) and rapid alternating movement speed by 23% (*t* = 4.58, df = 7, *P*=0.003, Figure 2(b)). There was also a significant improvement (60%) in the gait score after dynamic cycling (*t* = 3.15, df = 7, *P*=0.012, Figure 2(b)). These findings suggest that both amplitude/speed of hand movement and gait improve after dynamic cycling.

### 3.3. Mobility Assessment (Timed Up and Go)

There was a significant group-by-time interaction (*F* = 3.12, df = 7, *P*=0.005) for Timed Up and Go (TUG) (Figure 2(c)). Bonferroni correction was set to *P* ≤ 0.006(0.05/8) to account for paired samples *t*-test comparison. After this correction, there were significant improvements in the high-cadence cycling group between pretesting and session 5 (*t* = 4.140, df = 7, *P*=0.004), session 6 (*t* = 4.410, df = 7, *P*=0.003), and post (48 hours after the last cycling session, *t* = 4.617, df = 7, *P*=0.002). TUG time in the dynamic cycling group improved by 13% (1.17 s), compared to a 3% worsening in the control group, from pretesting to posttesting. The control group showed no significant improvements between sessions.

## 4. Discussion

This study demonstrates that six 30-minute bouts of high-cadence cycling improve motor function, gait, and mobility in individuals with idiopathic PD. Sustained improvements in motor function, gait, and mobility were seen immediately after high-cadence cycling. Additionally, participants in the high-cadence cycling group continued to show improvements in the overall UPDRS motor score, gait, and mobility beyond three cycling sessions.

Improvement in overall UPDRS Part III Motor score from baseline to posttesting improved by 17% or 2.5 points. Interestingly, participants were still receiving additional improvements in the overall UPDRS score past three exercise sessions. This change is within the minimum clinically important difference (2.3–2.7 points) as reported by Shulman et al. [27]. These findings are similar with previous studies, which showed that three 1-hour sessions of dynamic high-cadence cycling lead to a 13% improvement (2.5 pts) on the UPDRS Part III Motor exam [13]. Moreover, eight weeks of forced (high-cadence) exercise (FE) on a tandem bicycle significantly resulted in a 35% improvement in UPDRS Part III Motor score [14]. A detailed examination of components of the UPDRS Motor test showed that rigidity and bradykinesia measures showed the greatest change and that these changes occurred in both the lower and upper extremity. Both movement amplitude and speed improved after six bouts of high-cadence cycling. In addition, rapid alternating hand movement speed increased by 23% from baseline testing to posttesting in the high-cadence cycling group. Hand movement amplitude and rapid alternating hand movement speed may have improved due to motor learning and improvements in motor timing [28, 29].

In addition to upper-body motor function improvements, six sessions of high-cadence cycling also improved gait and mobility. These findings are consistent with other work that has shown increased gait velocity after high-cadence cycling [30, 31] as well as improvements in the TUG test [13]. The minimal detectable change (MDC) for the TUG test in individuals with Parkinson's disease is 3.5 s [32]. Although our reported change is only 1.7 s, the high-cadence cycling group showed a baseline TUG completion time of 10.1 s which is faster than the previous reported values in a similar population (10.4 s) [33]. Therefore, there is likely a ceiling effect on the potential improvement after high-cadence cycling in these subjects. Further examination of the potential of this intervention to improve mobility is warranted in a population at greater risk of falling and those with mobility deficits.

These results show that there is a sustained improvement in bradykinesia in the upper and lower extremity, gait, and mobility. Motor learning and improvements in motor timing may drive these changes over time. The basal ganglia is involved in perceiving the timing of external events and adjusting the temporal component of movements appropriately in response to task demands. Timing deficits are common problems in individuals with PD, as demonstrated by reduced movement speed and delayed movement onset. Even with levodopa medication, individuals with PD show poor timing control, as evidenced by inability to adjust movement timing to an external stimulus, compared with nondisabled adults. Therefore, exercise interventions should target these timing deficits and related cortico-basal ganglia circuitry to optimize motor benefit. A critical feature of high-cadence cycling (and other high-velocity training interventions) is an emphasis on timing/speed of pedaling frequency, using a motor, without increasing fatigue. Furthermore, the upper extremity benefit from a lower body exercise intervention is important for individuals with PD that may not be able to do upper extremity exercise due to severe tremor or rigidity.

Lastly, these findings show that individuals with PD are able to participate in and gain benefit from a high-cadence cycling-based intervention and that these benefits are maintained 48 hr after a cycling bout. While this does not directly answer the question regarding the optimal frequency of training, it does provide comparative data for longer intervention periods as well as direct comparisons of more or less frequent high-cadence cycling bouts.

There are a few limitations to this study. First is the small sample size and the heterogeneous study population. There was some variation in the cycling performance variables (torque) between subjects in the cycling group. Some individuals were able to keep up with the motor speed better than others. Despite the large standard deviations, there were still significant differences in improvements between the two groups (cycling and stretching). Future studies should examine if these patterns are consistent in a larger population with PD. It is also important to note that the participants in this study showed lower baseline UPDRS III scores (14.2) compared to similar studies by Ridgel et al. (baseline UPDRS score of 28) [13, 14]. Previous studies have suggested that individuals with greater motor deficits will show greater improvement after bouts of high-cadence cycling.

## 5. Conclusion

This study suggests that individuals with PD can complete six successive sessions of high-cadence cycling and that improvements in measures of bradykinesia and mobility are maintained at least 48 hours after training is complete. This suggests that exercise interventions should include elements that focus on timing and speed to promote changes in motor circuitry. Therefore, future studies should examine changes in motor timing and brain-based changes to investigate potential mechanisms of these changes.

## Figures and Tables

**Table 1:** Participant demographics.

Demographics	Dynamic cycling (*N*=8)	Stretching (*N*=8)	*P* value
Age (y)	69.9 ± 7.4	70.0 ± 6.4	0.972
Gender (M/F)	4/4	5/3	—
Hoehn and Yahr (H&Y)	1.4 ± 0.52	1.9 ± 0.35	0.043^*∗*^
Height (cm)	173.3 ± 6.1	175.6 ± 10.0	0.602
Weight (kg)	77.6 ± 9.5	94.3 ± 19.2	0.044^*∗*^
Physical activity (steps/day)	4,096.18 ± 2,995.9	4,207.26 ± 3,115.5	0.366
Duration of PD (y)	4.50 ± 1.6	6.38 ± 2.56	0.101
UPDRS baseline	14.13 ± 2.1	14.38 ± 3.5	0.866

Values represent mean ± standard deviations. ^*∗*^*P* values <0.05, independent samples *t*-test.

## Data Availability

The outcome data used to support the findings of this study are available from the corresponding author upon request.

## References

[1] Kowal S. L., Dall T. M., Chakrabarti R., Storm M. V., Jain A. (2013). The current and projected economic burden of Parkinson’s disease in the United States. *Movement Disorders*.

[2] Marras C., Beck J. C., Bower J. H. (2018). Prevalence of Parkinson’s disease across North America. *npj Parkinson’s Disease*.

[3] Schenkman M., Moore C. G., Kohrt W. M. (2018). Effect of high-intensity treadmill exercise on motor symptoms in patients with de novo Parkinson disease. *JAMA Neurology*.

[4] Prodoehl J., Rafferty M. R., David F. J. (2014). Two-year exercise program improves physical function in Parkinson’s disease. *Neurorehabilitation and Neural Repair*.

[5] Sajatovic M., Ridgel A., Walter E. (2017). A randomized trial of individual versus group-format exercise and self-management in individuals with Parkinson’s disease and comorbid depression. *Patient Preference and Adherence*.

[6] Silva-batista C., Corcos D. M., Roschel H. (2016). Resistance training with instability for patients with Parkinson’s disease. *Medicine & Science in Sports & Exercise*.

[7] Hashimoto H., Takabatake S., Miyaguchi H., Nakanishi H., Naitou Y. (2015). Effects of dance on motor functions, cognitive functions, and mental symptoms of Parkinson’s disease: a quasi-randomized pilot trial. *Complementary Therapies in Medicine*.

[8] Frazzitta G., Maestri R., Ghilardi M. F. (2013). Intensive rehabilitation increases BDNF serum levels in parkinsonian patients. *Neurorehabilitation and Neural Repair*.

[9] van Nimwegen M., Speelman A. D., Overeem S. (2013). Promotion of physical activity and fitness in sedentary patients with Parkinson’s disease: randomised controlled trial. *BMJ*.

[10] Alberts J. L., Phillips M., Lowe M. J. (2016). Cortical and motor responses to acute forced exercise in Parkinson’s disease. *Parkinsonism & Related Disorders*.

[11] Ridgel A. L., Abdar H. M., Alberts J. L., Discenzo F. M., Loparo K. A. (2013). Variability in cadence during forced cycling predicts motor improvement in individuals with Parkinson’s disease. *IEEE Transactions on Neural Systems and Rehabilitation Engineering*.

[12] Ridgel A. L., Peacock C. A., Fickes E. J., Kim C.-H. (2012). Active-assisted cycling improves tremor and bradykinesia in Parkinson’s disease. *Archives of Physical Medicine and Rehabilitation*.

[13] Ridgel A. L., Phillips R. S., Walter B. L., Discenzo F. M., Loparo K. A. (2015). Dynamic high-cadence cycling improves motor symptoms in Parkinson’s disease. *Frontiers in Neurology*.

[14] Ridgel A. L., Vitek J. L., Alberts J. L. (2009). Forced, not voluntary, exercise improves motor function in Parkinson’s disease patients. *Neurorehabilitation and Neural Repair*.

[15] Fisher B. E., Li Q., Nacca A. (2013). Treadmill exercise elevates striatal dopamine D2 receptor binding potential in patients with early Parkinson’s disease. *Neuroreport*.

[16] Fisher B. E., Wu A. D., Salem G. J. (2008). The effect of exercise training in improving motor performance and corticomotor excitability in people with early Parkinson’s disease. *Archives of Physical Medicine and Rehabilitation*.

[17] Petzinger G. M., Fisher B. E., McEwen S., Beeler J. A., Walsh J. P., Jakowec M. W. (2013). Exercise-enhanced neuroplasticity targeting motor and cognitive circuitry in Parkinson’s disease. *The Lancet Neurology*.

[18] Mohammadi-Abdar H., Ridgel A. L., Discenzo F. M., Phillips R. S., Walter B. L., Loparo K. A. (2016). Test and validation of a smart exercise bike for motor rehabilitation in individuals with Parkinson’s disease. *IEEE Transactions on Neural Systems and Rehabilitation Engineering*.

[19] Mohammadi Abdar H., Ridgel A., Discenzo F., Loparo K. (2015). Design and development of a smart exercise bike for motor rehabilitation in individuals with Parkinson’s disease. *IEEE/ASME Transactions on Mechatronics*.

[20] Barkley J. E., Glickman E., Fennell C., Kobak M., Frank M., Farnell G. (2018). The validity of the commercially-available, low-cost, wrist-worn movband accelerometer during treadmill exercise and free-living physical activity. *Journal of Sports Sciences*.

[21] Ault D. The effects of dynamic cycling on motor function, gait, mobility and balance in individuals with Parkinson’s disease. https://etd.ohiolink.edu/.

[22] Borg G. (1998). *Borg’s Perceived Exertion and Pain Scales*.

[23] Goetz C. G. (2008). *MDS-UPDRS Training Program & Exercise*.

[24] Heldman D. A., Espay A. J., LeWitt P. A., Giuffrida J. P. (2014). Clinician versus machine: reliability and responsiveness of motor endpoints in Parkinson’s disease. *Parkinsonism & Related Disorders*.

[25] Mera T. O., Heldman D. A., Espay A. J., Payne M., Giuffrida J. P. (2012). Feasibility of home-based automated Parkinson’s disease motor assessment. *Journal of Neuroscience Methods*.

[26] Shumway-Cook A., Brauer S., Woollacott M. (2000). Predicting the probability for falls in community-dwelling older adults using the timed up & go test. *Physical Therapy*.

[27] Shulman L. M., Gruber-Baldini A. L., Anderson K. E., Fishman P. S., Reich S. G., Weiner W. J. (2010). The clinically important difference on the unified Parkinson’s disease rating scale. *Archives of Neurology*.

[28] Jones C. R. G., Jahanshahi M. (2014). Motor and perceptual timing in Parkinson’s disease. *Advances in Experimental Medicine and Biology*.

[29] Wu T., Hallett M., Chan P. (2015). Motor automaticity in Parkinson’s disease. *Neurobiology of Disease*.

[30] Stuckenschneider T., Helmich I., Raabe-Oetker A., Froböse I., Feodoroff B. (2015). Active assistive forced exercise provides long-term improvement to gait velocity and stride length in patients bilaterally affected by Parkinson’s disease. *Gait & Posture*.

[31] McGough E. L., Robinson C. A., Nelson M. D. (2016). A tandem cycling program. *Journal of Neurologic Physical Therapy*.

[32] Huang S.-L., Hsieh C.-L., Wu R.-M., Tai C.-H., Lin C.-H., Lu W.-S. (2011). Minimal detectable change of the timed “up & go” test and the dynamic gait index in people with Parkinson disease. *Physical Therapy*.

[33] Schenkman M., Ellis T., Christiansen C. (2011). Profile of functional limitations and task performance among people with early- and middle-stage Parkinson disease. *Physical Therapy*.

